# Numerical Modeling of Chloride Transport in Concrete under Cyclic Exposure to Chloride

**DOI:** 10.3390/ma15175966

**Published:** 2022-08-29

**Authors:** Jae-Min Lee, Sung In Hong, Hee Jun Yang, Dong-Hyuk Jung

**Affiliations:** 1 Department of Civil Engineering, Kumon National Institute of Technology, Daehak-ro 61, Gumi 39177, Korea; 2 Environment and Energy Research Team, Hyundai Steel Company, Bukbusaneop-ro 1480, Dangjin 31719, Korea; 3 R&D Team, Bricon Lab Inc., Advanced Construction Materials Testing Center, Dalgubeol-dearo 1095, Daegu 42601, Korea; 4 Department of Highway & Transportation Research, Korea Institute of Civil Engineering and Building Technology, Goyangdae-ro 283, Ilsanseo-gu, Goyang 10223, Korea

**Keywords:** cyclic exposure, chloride penetration, pore size distribution, moisture level

## Abstract

Concrete structures under cyclic exposure to chlorides entail a higher risk of embedded steel corrosion along with accelerated ionic ingress from the environment. This study proposes a coupled transport model considering moisture and chloride distribution in concrete to investigate the influence of a cyclic exposure condition on chloride penetration. In this model, pore size distribution to quantify the effective pore space for moisture and chloride mobilizations was determined to establish the governing equation for chloride transport through non-saturated concrete. From the simulation results, the rate of chloride penetration increases with decreasing ambient humidity levels due to the enhanced chloride convection. Finally, the coupled transport model was verified by comparing in-situ data, showing reasonable correlations with 0.83 and 0.93 of determinant coefficients for 22 and 44 years of exposure, respectively, while those obtained from LIFE 365 were much lower.

## 1. Introduction

A large number of steel-reinforced concrete structures are located in saline environments that directly threaten structural safety in terms of chloride-induced corrosion. Despite the protective layer on the reinforcement and inhibitive nature of cement compounds to corrosion at the steel–concrete interface [1], concrete as a porous material continuously permits chloride sources from its surrounding environments. After long-term exposure to chlorides, corrosion of steel in concrete may take place to impose structural damages via serial volume expansions of the corrosion products, leading to an increase in the rate of species ingress by cracking, and the load-carrying capacity of steel is subsequently reduced due to its cross-section loss [2,3,4,5]. In order to secure the required service life of steel-reinforced concrete structures at minimum cost, a proper prediction model based on both the concrete properties and environmental actions is in need.

Several experimental studies investigating the chloride penetration in concrete under cyclic exposure condition have been carried out [6,7], revealing that the rate of ionic penetration could be accelerated by moisture flux within concrete. The additional driving force to enhance the rate of chloride transport in concrete is due to convection with moisture transport, which then incorporates diffusion within continuous water channels in pores [8]. Thus, a cyclic exposure condition could impose a higher risk of chloride-induced corrosion in concrete by the combined transport phenomenon than a submerged state alone [9]. 

Many modeling works based on a semi-empirical relationship between moisture level and chloride diffusion coefficient have been carried out in predicting chloride transport in concrete under a cyclic exposure condition [10,11,12,13,14]. In these approaches, Saetta et al. [10] suggested moisture diffusivity as a function of relative humidity to establish a chloride diffusivity, considering the effects of moisture variation in concrete on the ionic transport rate. However, moisture content obtained from vapor adsorption or desorption at a given humidity level highly changes with pore size distribution depending on mix condition [15], which directly influences the moisture transport rate [16], thereby affecting chloride penetration at non-saturated conditions. On the other hand, considering the pore size distribution in concrete, the statistical permeability model used for defining the moisture permeability in porous material has often been used to predict the chloride ingress under cyclic exposure conditions [17,18,19]. Nevertheless, a simple expression to the tortuosity in terms of porosity never achieves the value at cement paste above 4.0 [18], which would otherwise largely control both the transport rates of moisture [16] and chloride ions [20]. Furthermore, pore size distribution with a single peak [19] may be insufficient in analyzing the moisture distribution in concrete with a wide range of pore sizes, which would affect the chloride penetration through the non-saturated pore network. 

Several empirical studies on determining the chloride diffusivity in non-saturated concrete as a function of moisture level were carried out for a practical purpose. Based on the moisture-dependent chloride diffusivity proposed by Saetta et al. [10], Nielsen and Geiker [21] developed it to reflect the degree of saturation for each pore component on the chloride diffusion at partial saturation. Meanwhile, Climent et al. [22] set up a new experimental methodology for determining the moisture-dependent chloride diffusivity using a gaseous chloride source to penetrate partially saturated concrete. Based on this result, Vera et al. [23] found a more accurate S-shaped relationship between chloride diffusivity and moisture level according to the chloride binding effect. Moreover, Olsson et al. [24] proposed a way to derive the chloride diffusivity at non-saturated concrete by applying measured conductivity to the Nernst–Einstein equation. However, those approaches are only valid in a constant moisture condition without considering chloride convection with moisture flow in concrete.

For a practical purpose, this paper assessed chloride contamination in concrete under cyclic exposure conditions using the coupled transport model for both moisture and chloride to improve the accuracy of service life prediction of the reinforced concrete structures linked with chloride-induced corrosion. In quantifying the rate of mass transport in a porous medium, pore size distribution was firstly determined and used to derive connected pore fraction and tortuosity for both moisture and chloride mobilizations. Then, moisture distribution in concrete under thermodynamic conditions was calculated using permeation models for liquid and vapor phases through saturated and non-saturated pores. Based on this, chloride penetration in concrete, depending on moisture distribution, could be modeled to predict the time to onset of steel corrosion at assumed cover depth. Consequently, chloride diffusion coefficient and surface chloride concentration as functions of exposure time and ambient humidity level were determined by fitting a diffusion model to the numerical solutions in order to evaluate diffusion behaviors under cyclic exposure conditions. The proposed model was verified by comparing the results of in situ observation with those obtained from LIFE 365.

## 2. Modelling the Mass Transport in the Cement Matrix

### 2.1. Pore Size Distribution

The process of mass transport in hydrated cement paste (HCP) is primarily dependent on the pore size distribution that changes with the cement hydration, where the hydration rate is largely affected by the initial water to cement ratio (*w*/*c*) [25]. As the cement hydration proceeds, connected pore space decreases to slow down the process of mass transport. The nature of the pore structure in the numerical prediction of mass transport in HCP was reflected using some semi-empirical models developed in previous studies [26,27,28] for estimating the degree of cement hydration and resultant modification of the pore size distribution.

Usually, the size of pores to affect the transport process in HCP is less than 5000 nm in radius [2,25], over which most of the pores are entrapped or even isolated. Hence, in this study, the pore size distribution used for simulating the moisture and chloride ion transport was determined based on the three types of pore components with different size specifications, as listed in Table 1. All pores were assumed to be cylindrical and partially interconnected to each other. The extended formula of the Rayleigh–Ritz (R–R) density function [16] was used to express a normalized pore size distribution in HCP including gel pores and small and large capillary pores such that

(1) $$f\left(r\right)=\frac{1}{\emptyset_{t}}\overset{3}{\underset{i=1}{\sum }}\emptyset_{i}B_{i}exp\left(-B_{i}r\right)\;$$

where Ø_t_ is the total porosity, *i* is the index of pore component corresponding to gel pores (*i* = 1), small capillary pores (*i* = 2), and large capillary pores (*i* = 3), Ø*_i_* is the porosity at *i*, *B_i_* is the parameter of R–R density function at *i*, and *r* is the pore radius (m). To consider the effects of changing volume fraction of phases with time on the densification of HCP, Power’s model [29] was adopted herein to calculate the porosity values in Equation (1) such that

(2) $$\emptyset_{1}=\frac{0.19\alpha }{w/c+0.32}\;$$

(3) $$\emptyset_{ca}=\emptyset_{2}+\emptyset_{3}=\frac{w/c-0.36\alpha }{w/c+0.32}\;$$

where *w*/*c* is the water to cement ratio, *α* is the degree of hydration, and Ø*_ca_* is the capillary porosity. The porosity value for the small and large capillary pores is determined by applying a ratio (*m*) to the capillary porosity (i.e., $\emptyset_{2}=m\times \emptyset_{ca},\;\emptyset_{3}=\left(1-m\right)\times \emptyset_{ca}$). In Yamaguchi’s study [30], the pores with a radius over 100 nm were presumed to be partially connected to the ‘capillary transport pores’ corresponding to the small capillary pores, and the author also proposed the parameter (*m*) defined as the volume ratio of the ‘capillary storage pores’ corresponding to the large capillary pores to the total capillary pores. This linear relationship between the capillaries was also verified by finding the closest curve of sorption isotherm to the one based on the BSB model suggested by Huang et al. [16], who originally intended to determine the pore parameters in Equation (1). In this study, *m* was set as a single regression parameter and optimized to be 0.75 under the conditions of *w*/*c* (0.3–0.6) with curing periods up to 500 days, for which age means a fully hydrated condition.

The porosity of each pore component in the pore size distribution varying with time and *w*/*c* was determined using the cement hydration model with four rate coefficients consisting of formation and destruction of the initial impermeable layer over cement particles, the activated chemical reaction process, and the following diffusion-controlled process [26,27,28]. In this approach, the rate of cement hydration is considered to be decreasing as the contact area of cement particles and water (*S_w_*) decreases and can be expressed as [31]

(4) $$\frac{d\alpha }{dt}=\frac{3\left(S_{w}/S_{0}\right)\rho_{w}C_{w-free}}{\left(v+w_{g}\right)r_{0}\rho_{c}}\frac{1}{\left(\frac{1}{k_{d}}-\frac{r_{0}}{D_{w}}\right)+\frac{r_{0}}{D_{w}}{\left(1-\alpha \right)}^{-1/3}+\frac{1}{k_{r}}{\left(1-\alpha \right)}^{-2/3}}$$

(5) $$k_{d}=\frac{B_{ce}}{\alpha^{1.5}}+C_{ce}\alpha^{3}$$

(6) $$D_{w}=D_{w0}\ln\left(\alpha^{-1}\right)$$

(7) $$C_{w-free}=\frac{w/c-0.4\alpha }{w/c}$$

where *t* is the time, *v* is the stoichiometric ratio by mass of *w*/*c* (=0.25), *w*_g_ is the physically bound water in the CSH gel (=0.15), *ρ_w_* is the density of water, *ρ_c_* is the density of cement equal to 3200 kg/m^3^, *C_w-free_* is the amount of water at exterior of the CSH gel, *r*_0_ is the average radius of unhydrated cement particles, which was assumed to be 6.18 um, *S*_0_ is the total surface area of cement particles, *B*_ce_ and *C*_ce_ are the rate coefficient for controlling the initial shell formation and destruction, *D*_w0_ is the initial value of the effective water diffusion coefficient through the CSH gel, and *k*_r_ is the coefficient of reaction rate of cement. The fraction of effective surface area for cement hydration (i.e., $S_{w}/S_{0}$) is a function of *α* and *w*/*c*, determined based on the calculation procedure for the geometrical formulation of a cement particle in a unit cell [26]. 

Along with the cement hydration, the capillary pores in HCP become filled with the hydration products, thereby increasing the space of gel pores and decreasing that of capillary pores. Consequently, the mean size of capillary pores becomes reduced and approaches that of gel pores to decrease the overall connectivity of pores in HCP. In this respect, the critical radius (*r_c_*) as a decisive factor to the mass transport [17] is introduced as a pore parameter (i.e., 1/*B*_2_) in the pore size distribution in Equation (1). Based on the simulation result from the microstructure model proposed by Garboczi and Bentz [32], this study suggested a similar form of equation determining *r*_c_ as a function of capillary porosity such that

(8) $$r_{c}=r_{c0}+H\left(\emptyset_{ca}-\emptyset_{c0}\right)\times a\times {\left(\emptyset_{ca}-\emptyset_{c0}\right)}^{2}$$

where *r_c_*_0_ and Ø*_c_*_0_ are the pore radius and capillary porosity at the end of the percolation of capillary pores, and *H*(*x*) is the Heaviside step function denoting 0 at x≤0 and 1 at x>0. *r_c_*_0_ was set as 10.5 nm [17], at which Ø*_ca_* is equal to Ø*_c_*_0_. On the other hand, *B*_1_ and *B*_3_ in Equation (1) were assumed to be constant, irrespective of the w/c and cement hydration, as given in Table 1. Then, 1/*B*_1_ is the mean size of gel pores and 1/*B*_2_ is the one derived from the different classifications of the large capillary pores [17], at which the prediction of moisture transport in HCP is not significantly affected.

### 2.2. Tortuosity of Pores

The tortuosity factor (*τ*) should be considered to evaluate the resistance to the mass transport quantitatively as an actual flow path in a cement pore system. In a previous study [33], Kats and Thompson permeability theory with a calculated pore size distribution was used to determine *τ* by assuming that the cement pore system follows the Nernst–Einstein relation:

(9) $$\frac{\emptyset_{t}}{\tau }=\frac{D_{e}}{D_{0}}=\frac{\sigma_{e}}{\sigma_{0}}$$

where *D_e_* is the effective chloride diffusivity, *D*_0_ is the diffusion coefficient in a free liquid and taken as 2.05 × 10^−^^9^ m^2^/s at 20 °C of water temperature, and *σ_e_* and *σ*_0_ are the electrical conductivity in a cement matrix and free liquid. In this case, *D_e_* and *τ* in Equation (9) were computed on the basis of the simulated result obtained from the pore size distribution model in Equation (1), which could reflect the cement hydration process depending on the *w*/*c*.

### 2.3. Moisture Distribution

The moisture transport was simulated to determine the convective flux of chloride ions through the HCP under cyclic exposure conditions. By assuming that Kelvin’s thermodynamic equilibrium condition is maintained in the HCP, the following partial differential equation for describing the moisture transport can be derived [16]:

(10) $$\frac{\partial w}{\partial h}\frac{\partial h}{\partial t}=\frac{\partial }{\partial x}\left(K_{l}\frac{\rho_{w}RT}{M_{w}h}\frac{\partial h}{\partial x}+K_{v}P_{0}\frac{\partial h}{\partial x}\right)$$

where *w* is the moisture content (kg/m^3^), *h* is the relative humidity, *t* is the time (sec), *x* is the distance (m), *K_l_* is the permeability of the liquid water (kg/m∙s∙Pa), *ρ_w_* is the density of water (kg/m^3^), *R* is the gas constant (J/kg∙K), *T* is the temperature (K), *M_w_* is the molecular weight of water (kg/mol), *K_v_* is the permeability of water vapor (kg/m∙s∙Pa), and *P_0_* is the saturation vapor pressure (Pa). The *K_l_* was determined based on the statistical permeability theory [17], considering a probabilistic approach on the pore size distribution under saturated pore space such that

(11) $$K_{l}=\frac{\rho_{w}\emptyset_{t}^{2}}{8\tau^{2}\eta }{\left(\int_{0}^{r_{s}}rf\left(r\right)dr\right)}^{2}$$

where *η* is the viscosity of liquid water (Pa∙s), accounting for 0.001 Pa∙s at 20 °C, and *r*_s_ is the radius below which pores are saturated (m). The water vapor transports only through the air-filled space with a partially saturated condition. Based on the Knudsen diffusion model, *K_l_* can be expressed as [13]

(12) $$K_{v}=\frac{D_{v}\left(1-S\right)}{\tau \left[1+l_{m}/2\left(r_{m}-t_{a}\right)\right]}\left(\frac{M_{w}}{RT}\right)$$

where *S* is the degree of saturation, *l_m_* is the mean free path of a water molecule (m) accounting for 10^−7^ m [34], *r_m_* is the mean size of pores over non-saturated spaces (m), and *D*_v_ is the diffusivity of water vapor in free space (m^2^/s). The parameters in Equations (10)–(12) were given in the previous studies [17,34].

(13) $$D_{v}=D_{v,ref}\frac{P_{ref}}{P_{0}h}{\left(\frac{T}{T_{ref}}\right)}^{1.88}$$

where, *D*_v_,_ref_ is the vapor diffusivity at reference pressure (*P_ref_*) and temperature (*T_ref_*) (*D_v_*_, *ref*_ = 21.6 × 10^−6^ m^2^/s, *P_ref_* = 11,325 Pa, *T_ref_* = 273.16 K) [34]. The mean radius for non-saturated pores was determined using the pore size distribution such that

(14) $$r_{m}=\frac{\int_{r_{s}}^{r_{max}}rf\left(r\right)dr}{\int_{r_{s}}^{r_{max}}f\left(r\right)dr}$$

where *r_max_* is the maximum pore radius (*m*), assumed to be 5 µm. In this study, moisture content associated with the vapor adsorption through non-saturated pores was excluded in the analysis since a very thin water channel below around 1.2 nm forms on the pore wall, at which the ionic transport can be considerably limited [34]. In addition, hysteric behavior between vapor adsorption and desorption on *S* was taken into account by using the following equations such that [17]

(15) $$S=S_{w}=\int_{0}^{r_{s}}f\left(r\right)dr$$

for wetting and

(16) $$S=S_{d}=S_{w}\left(1-ln\left(S_{w}\right)\right)$$

for drying, respectively. For a practical purpose, both saturation functions given in Equations (15) and (16) were used to represent the arbitrary moisture distribution in concrete under a cyclic exposure condition.

### 2.4. Chloride Transport

Under cyclic exposure to chloride, ionic diffusion via concentration gradient and convection with moisture flow simultaneously occurs through non-saturated pores. When considered both chloride diffusion and convection, the unidirectional chloride transport in concrete can be expressed as [11]

(17) $$\left(\frac{\partial C_{b}}{\partial C_{f}}+S\emptyset_{t}\right)\frac{\partial C_{f}}{\partial t}=\frac{\partial }{\partial x}\left(S\emptyset_{t}D_{e}\frac{\partial C_{f}}{\partial x}\right)+\frac{\partial }{\partial x}\left(S\emptyset_{t}C_{f}D_{h}\frac{\partial h}{\partial x}\right)$$

where *C_f_* is the free chloride concentration in pore solution (kg/m^3^), *C_b_* is the bound chloride concentration in solid phase (kg/m^3^), and *D_h_* is the moisture diffusion coefficient (m^2^/s), which accounts for the rate of combined liquid and vapor permeation as the following equation:

(18) $$D_{h}=\left(K_{l}\frac{\rho_{w}RT}{M_{w}h}+K_{v}P_{0}\right)\frac{\partial h}{\partial w}$$

Langmuir isotherm was used to describe a non-linear relationship between concentrations of free and bound chloride at equilibrium. The derivative formula of the relationship indicates the chloride binding capacity [11], which can be expressed as

(19) $$\frac{\partial C_{b}}{\partial C_{f}}=\frac{\alpha }{{\left(1+\beta C_{f}\right)}^{2}}$$

where *α* and *β* are the binding parameters, accounting for 1.14 and 0.18 respectively, deduced from the experimental data for OPC pastes with 0.4 *w*/*c* aged between 60 and 150 days [35]. 

Chloride ions in cement matrix are mostly transported through the capillary pores and partially in gel pore for which the diffusivity accounts for 400 times less than that of capillary [32], but the transport rate at gel pore could be dominated as the cement matrix become densified to reduce the capillary pore space. Considering the combined effects of gel and capillary pores on the chloride diffusion with the determined tortuosity assumed to be equal for both pore systems, a composite model [36] was used to determine the effective chloride diffusivity such that

(20) $$D_{e}=\frac{y\left(\emptyset_{2}+\emptyset_{3}\right)D_{0}}{\tau^{2}}+\frac{\left(1-y\right)\left(\emptyset_{2}+\emptyset_{3}\right)+\emptyset_{1}}{\tau^{2}}\left(\frac{\left(1-y\right)\left(\emptyset_{2}+\emptyset_{3}\right)+\emptyset_{1}}{\left(\frac{\left(1-y\right)\left(\emptyset_{2}+\emptyset_{3}\right)}{D_{0}}+\frac{\emptyset_{1}}{D_{0}/400}\right)}\right)$$

where *y* is the percolating fraction for the diffusive phase, which in this study was determined by equalizing the probability of moisture entrapment over the entire pore network (i.e., $\left(S_{d}-S_{w}\right)/S_{d}$) as deduced from the virgin wetting and drying isotherms to that of non-diffusive ionic path such that

(21) $$y=1-\int_{h_{min}}^{h_{max}}\frac{S_{d}-S_{w}}{S_{d}}dh$$

where *h_min_* and *h_max_* are relative humidity values at completely dried and saturated conditions, respectively. Accordingly, *D_e_* in Equation (20) can take into account both effective pore fraction of chloride diffusion at capillary pore and mixed diffusive phase combined by percolating gel pore and non-percolating capillary pore (i.e., end or ink-bottle pores).

### 2.5. Boundary Conditions

This study used 12 h per day for each wet/dry cycle to reflect cyclic exposure conditions to the concrete surface in a typical tidal/splash zone. As for the moisture flux into or out of the concrete surface, the following boundary condition [37] was used such that

(22) $$D_{h}\frac{\partial h}{\partial x}=B_{h}\left(h_{s}-h_{en}\right)$$

where *B_h_* is the mass transfer coefficient for relative humidity (m/s) accounting for 4.05 × 10^−7^ m/s [37] and *h*_s_ and *h_en_* are the relative humidity at concrete surface and environment, respectively. During the wet condition, relative humidity at the concrete surface, *h_en_*, was assumed to be 99.5%, which is enough to achieve saturation (i.e., capillary saturation) but not for the supersaturation at 100% humidity level, since singularity in Kelvin’s equation occurs at this condition [18]. Chloride ion transfer through the material surface is achieved in the form of dissolved ions and depends on gradients of moisture and chloride concentrations. Accordingly, the following mixed boundary condition [10] was used to describe the chloride flux at the concrete surface such that

(23) $$D_{e}\frac{\partial C_{f}}{\partial x}=B_{c}\left(C_{s}-C_{en}\right)+C_{en}D_{h}\frac{\partial h}{\partial x}$$

where *B_c_* is the mass transfer coefficient for chloride ion (m/s), which is applicable at wetting, accounting for 1.0 × 10^−3^ m/s [38] and non-conductive at drying (i.e., *B_c_* = 0), and *C*_s_ and *C_en_* are the free chloride concentration (kg/m^3^) at the concrete surface and environment, respectively. *C_en_* was set as 0.5 M to mimic the concentration of chloride ions in typical seawater. The initial relative humidity in concrete was assumed to be 99.5% as a saturated condition, and no chloride contamination in concrete was assumed. Temperature was assumed to be 20 °C as a constant value in concrete for all the simulations. All the simulations for the chloride transport began with wetting and end with drying. 

### 2.6. Numerical Strategy

The Crank–Nicholson method was used to discretize the partial differential equations with 1 mm and 0.1 day of distance and time increment, respectively, to solve the numerical solution for the coupled transport model. The numerical oscillations resulting from the rapid change in boundary conditions with time were minimized using the Gauss–Seidel method, where the iterative calculation with a tolerance of 10^−6^ on the relative change in solutions was implemented. Before solving the chloride transport, analysis of moisture distribution was carried out to provide the spatial values (i.e., *h*, *S*, and *D_h_*), which are then applied to calculate chloride transport. Including the non-linear coefficients in the governing equations, values at the current time step derived from the spatial mass distribution at the previous time step were used to solve those at the next increment. This one step of iteration ends with achieving the specified convergence criteria (i.e., 10^−6^) and continues until values at all the time and distance nodes are determined. 

## 3. Chloride Transport in Non-Saturated Concrete 

### 3.1. Determination of Pore Structure 

**Figure 1:**
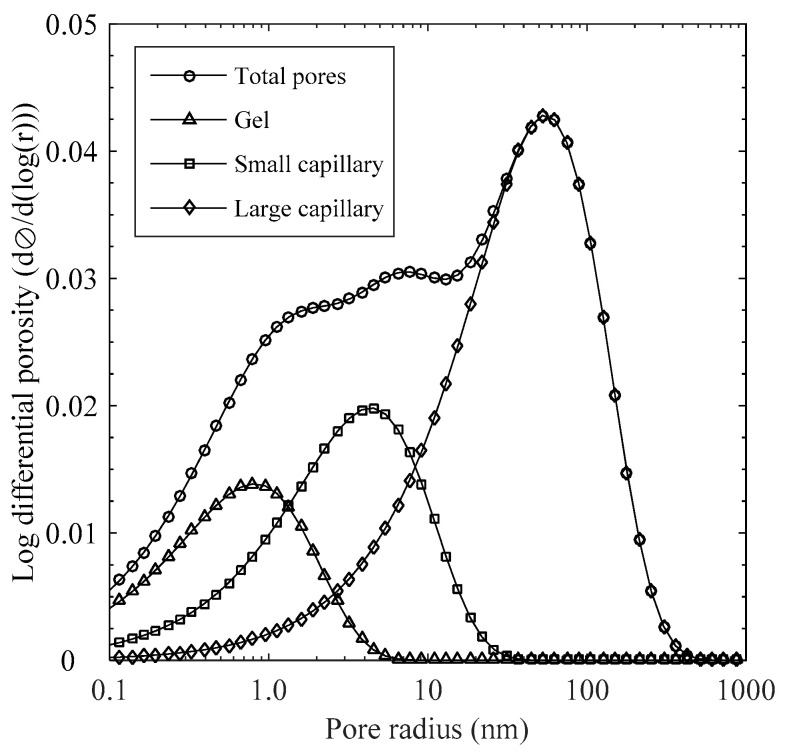
Calculated pore size distributions for each pore component together with overall pore size distribution.

To quantify transport rates of moisture and chloride in concrete, pore structure parameters were determined based on the pore size distribution and are given in Table 1. As seen in Figure 1, all types of pores are generally within the prescribed size ranges and mostly formed below 0.5 μm in radius, over which some inadvertent cracks during the sample preparation could be included in the MIP data [16]. The fraction of gel pore on total pore volume accounts for about 0.15 and mostly overlap with the small capillary pore up to about 0.01 μm while the large capillary pore has the highest contribution on the total porosity. Within the capillary size ranging from *r*_con_ (21.3 nm) to *r*_c_ (50.5 nm), the fraction of connected capillary pore (i.e., *F*(*r*_con_)) can be evaluated to determine the tortuosity. In general, smaller values of *r*_c_ and *F*(*r*_con_) indicate more tortuous and densified microstructure in terms of the species ingress into the concrete cover. 

**Figure 2:**
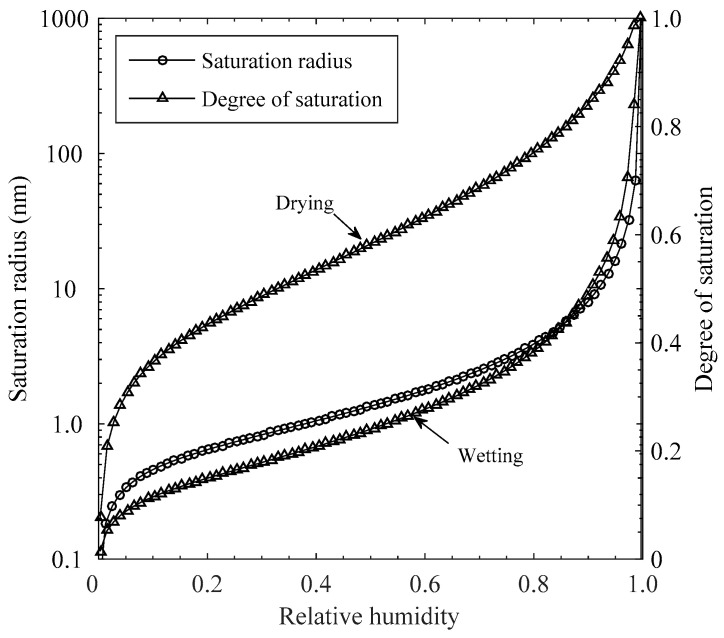
Moisture isotherms under drying and wetting together with saturation radius varying with relative humidity levels.

Simultaneously, under a thermodynamic equilibrium, the degree of saturation in concrete depending on relative humidity level was determined using the moisture isotherms for vapor adsorption and desorption, as shown in Figure 2. As relative humidity increases, saturation radius increases to raise overall water content with pore sizes up to 1000 nm, over which porosity increment is negligible. Also, the degree of saturation for vapor adsorption rapidly increases up to 1.0 nm in pore radius and gently with pore size up to about 4.0 nm, over which capillary condensation become again promoted due to the high portion of large capillary pore. The degrees of saturation under drying are consistently higher than those for vapor adsorption by the amount of entrapped water content where the pores bigger than saturation pore radius are partially saturated. Hence, under the cyclic exposure condition, a surplus amount of moisture due to the moisture entrapment under drying can resist a sudden drop of moisture level through the concrete cover. 

By applying the calculated degrees of saturation under drying and wetting to Equation (21), it is possible to obtain the percolating pore fraction for diffusive phase (i.e., *y*) and effective chloride diffusivity (i.e., *D_e_*), which are given in Table 1. In this case, *y* for the chloride diffusion considering the ink-bottle effect includes all types of pores and thus leads to a higher value than F(*r*_con_), which was determined through the only capillary pore and used to derive the tortuosity. 

### 3.2. Moisture Distribution under Wet/Dry Cycle

**Figure 3:**
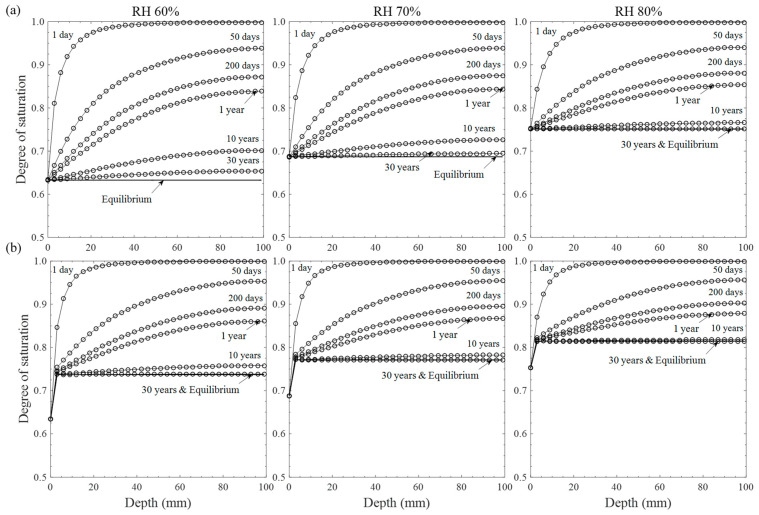
Moisture distributions with relative humidity levels under (**a**) constant drying and (**b**) 12 h for each wet/dry cycle.

A numerical solution for moisture transport was carried out on constant and wet/dry conditions to evaluate moisture distribution in concrete with a variation in ambient humidity levels (60–80%), as given in Figure 3. Degrees of saturation at all the concrete surfaces instantaneously equilibrate to the environments, accounting for 0.63, 0.68, and 0.75 at 60, 70, and 80% ambient humidity levels, respectively. According to the Figure, the rate of moisture evaporation from the inner part of the material to the drying surface decreases with time for both boundary conditions due to the reduction in moisture diffusivity, which is non-linearly proportional with moisture level [16,37]. After 10–30 years of exposure time, equilibrium in moisture level was mostly achieved through the interior of concrete, at which the moisture content becomes stabilized with time. As for the repeated wet/dry cycles indicated in Figure 3b, the rates of moisture convergence to equilibrium were much faster than those for the constant conditions due to the intermittent supply of water sources. After the moisture equilibrium, the degree of saturation through inner concrete depth increases from 0.73 to 0.82, corresponding to about 3.4 and 6.8 nm of saturation radius, as ambient humidity level increases from 60 to 80%. Hence, ionic transport in concrete can be enhanced or retarded by the external humidity level, which directly influences the formation of a water channel in pores for ionic diffusion [19].

### 3.3. Influence of Moisture Distribution on Chloride Ingress

**Figure 4:**
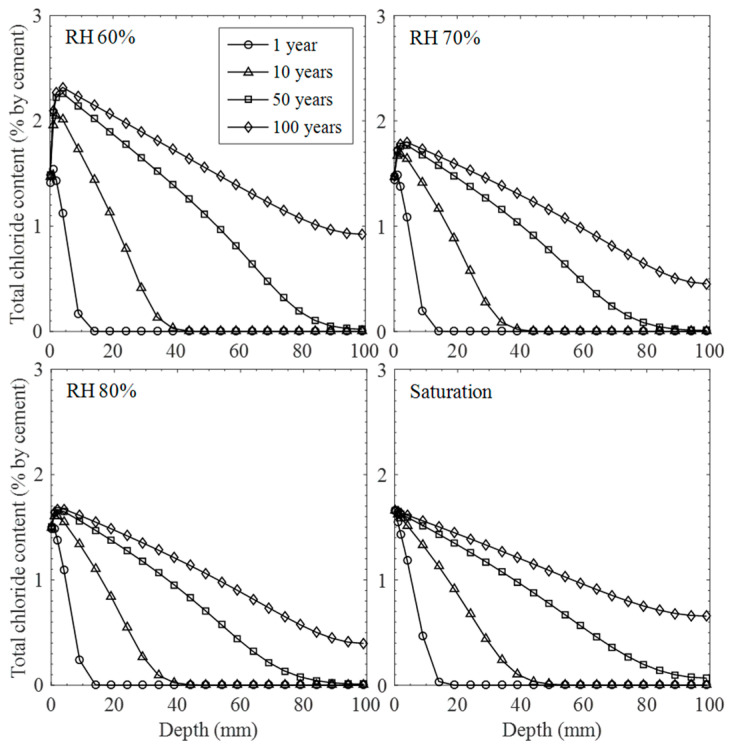
Time dependent concentration profiles for cyclic chloride exposure with different ambient humidity levels.

A numerical solution for the coupled transport model was implemented to investigate the effect of moisture conditions on the chloride transport in concrete. As given in Figure 4, chloride profiles at different ambient humidity levels were characterized by concentration evolution with time near the concrete surface, where the saturation condition indicates a constant surface concentration (1.65% by weight of cement). This different front behavior below saturation would be associated with the moisture-wicking with chloride source, originally defined as the moisture movement via liquid and vapor diffusion from a material surface contacted with a solution to the drying surface [39,40]. Under a cyclic exposure to chloride, the wick action in concrete could also occur during drying to draw moisture from the inner depth to the drying surface. Simultaneously, penetrated chloride contents recede from the concrete interior and mostly precipitate near the drying surface [40]. The maximum chloride contents for all the concentration profiles exist within 1–4 mm of the concrete surface, which could elevate the concentration gradient for diffusion into the interior of concrete depth. As indicated in Figure 3b, this specific positions for the chloride accumulation would arise from the repeated wet/dry cycles to generate the influential depth being affected with moisture intake and loss [41] (e.g., 3 mm in the present result). The concentration build-up near the surface substantially increases up to 10 years and is relatively gentle with further exposure time as the rate of moisture evaporation decreases with time to lower the wick action of chloride source from the inner depth. Particularly for the case of 60% ambient humidity level, the chloride build-up near the surface is always higher, consequently exceeding the surface concentration by about 1.5 times at 100 years. In addition, decreased ambient humidity level could increase the rate of chloride penetration during wetting as moisture gradient from the surface increases to promote the convective flow of chloride source into the depth. When compared with the saturation one, however, the rate of chloride transport with medium humidity levels is relatively low after long-term exposure, indicating that the concentration for the humidity level of 70% and 80% above 60 mm exceeds the value for saturation one at 100 years. This behavior is due to the lowered convection rate and the decreased diffusion rate with decreased moisture content, further discussed in the next paragraph.

**Figure 5:**
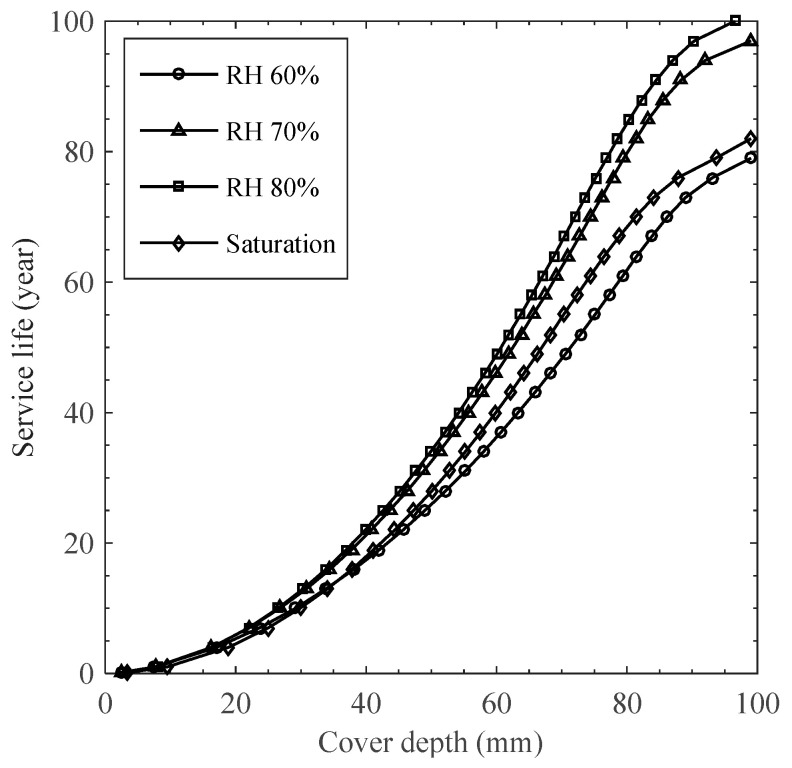
Expected service life dependent on cover depth and ambient humidity level according to the onset of corrosion of reinforcing steel.

As shown in Figure 5, chloride concentration at assumed cover depth up to 100 mm was calculated to investigate the influence of chloride penetration under cyclic exposure conditions on the service life estimation, determining the time of corrosion initiation on reinforcement. Since no certainty in defining the critical chloride concentration has been established, arising from the different chloride sources and several detection techniques [1], this study assumed the concentration at reinforcement as 0.4% by cement [42]. It can be seen that the difference in service life between humidity levels is marginal up to about 20 mm of cover depth and become higher away from the surface, leading to about 20 years of maximum difference at 100 mm of cover depth. As chloride penetrates deep into the concrete cover, moisture flux into or out of the inner depth decreases with decreasing moisture gradient, which will be marginal when the moisture equilibrium reaches over 10–30 years (see Figure 3b). Thus, the chloride penetration over the influential depth would be governed by the ionic diffusion at which the chloride diffusivity could increase with the volume of a water channel in pores [21,22,23,24]. When the moisture equilibrium occurs through the diffusion-controlled zone, water-filled porosity (denoted as *S*Ø*_t_*) decreases from 0.21 to 0.15 with decreasing ambient humidity level from the saturation to 60% (see Figure 3b). Despite the lowest water contents, however, service life for 60% RH is always minimized due to a large concentration gradient near the drying surface, which may enhance the overall transport rate even through a diffusion-controlled zone. Hence, it can be inferred from the results given in Figure 4 and Figure 5 that the chloride diffusion is a decisive factor in the high humidity region, while in low humidity level the overall transport rate would be governed by the convection effects: (1) permeation of chloride solution and (2) concentration build-up near the drying surface by repeated wet/dry cycles.

### 3.4. Expression for Chloride Diffusion in Non-Saturated Concrete

In most saline environments, concrete structures undergo cyclic exposures to chloride, which in turn causes non-linear properties in terms of both chloride diffusion coefficient and surface chloride concentration [43]. To evaluate chloride diffusion behavior under cyclic exposure conditions, diffusion parameters with functions of time and ambient humidity level were determined by fitting the numerical results to the diffusion model with a time-dependent boundary condition [44] such as:

(24) $$C_{t}\left(x,t\right)=\frac{2}{\sqrt{\pi }}\int_{\frac{x}{2\sqrt{D_{a}t}}}^{\infty }C_{s}\left(t-\frac{x^{2}}{4D_{a}\omega^{2}}\right)exp\left(-\omega^{2}\right)d\omega$$

where, *C_t_* is the total chloride content (% by weight of cement), *C_s_* is the surface chloride content (% by weight of cement), and *D_a_* is the apparent diffusion coefficient (m^2^/s), which was determined by the following equation [45]:

(25) $$D_{a}=D_{a,ref}{\left(\frac{t_{ref}}{t}\right)}^{m}$$

where, *D_a,ref_* is the reference chloride diffusivity (m^2^/s) at *t_ref_* (year), and *m* is the decay constant. In this study, obtained value from regression analysis (Equation (25)) was used as a constant value in Equation (24), assuming that change in concrete properties until the specific exposure time (*t)* is negligible. As for the expression of surface chloride concentration, this study adopted a logarithm function due to the higher accuracy of a fitting result as compared with other types (e.g., linear and square root) [46], which is given as:

(26) $$C_{s}=C_{0}+k\cdot ln\left(t\right)$$

where, *C*_0_ is the concentration at initial exposure time, assuming as a constant value (i.e., 1.65% by weight of cement) derived from the saturated condition, and k is the regression parameter. 

**Figure 6:**
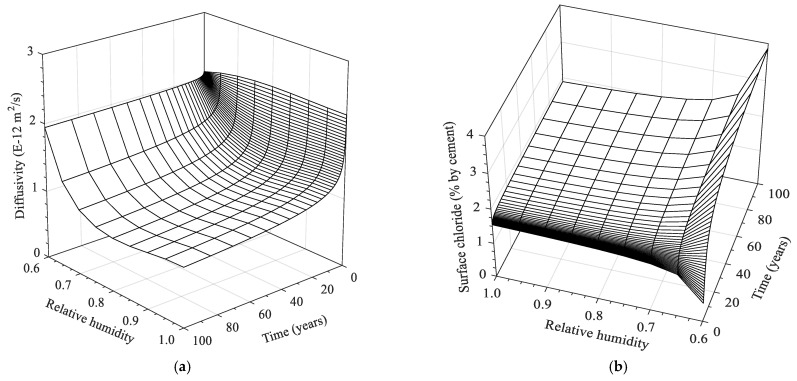
Change in parametric values dependent on relative humidity and time for (**a**) apparent diffusion coefficient and (**b**) surface chloride content.

By fitting Equations (24)–(26) to the numerical results of chloride penetration under the boundary condition described in Section 2.4, it was possible to obtain the regression parameters given in Equations (25) and (26). In this case, the initial time interval consists of 50, 100, and 200 days, followed by 1–5 years with one-year increments since a rapid change in diffusion parameters occurs within these periods [44,45,46]. After that, 5 years of time increments were used up to 100 years of total exposure time. The relative humidity for the boundary condition at drying ranges from 60% to the saturation with 5% increments. As the result of regression analysis, cubic and non-linear formulas as a function of relative humidity were obtained and expressed as:

(27) $$m=6.55\times h^{3}-17.72\times h^{2}+15.82\times h-4.52$$

for *D_a_* and

(28) $$k=\frac{h}{-27.13+47.30\times h}$$

for *C_s_*, respectively. Mean square errors between numerical and analytical solutions were 0.0083–0.0759, depending on the ambient humidity levels, as given in Table 2. Using Equations (25)–(28), chloride diffusion coefficient and surface chloride concentration, taking into account time dependency and ambient humidity level, can be determined, depicted in Figure 6.

As shown in Figure 6a, chloride diffusivity at the initial period up to 100 days slightly increases with humidity level. After that, it generally decreases with exposure time, which is more significant at higher humidity levels. The former might be related to the fact that less significance in convection effects at the initial wet/dry period leads to a similar behavior with the chloride diffusion at which the rate is proportionally dependent on the moisture level [21,22,23,24]. As for the latter, time-dependent diffusivity irrespective of moisture condition may be attributed to the cement hydration, causing pore refinement with time, though this factor was not reflected in this study. Thus, it was presumed that the decreasing trend of diffusivity with time mainly arises from the chloride binding capacity varied with concentration distribution [47], which would alter the system of chloride diffusion from a non-steady state into a near-steady state at continuous chloride contamination in concrete. Especially over 80% humidity levels, the chloride diffusivity is more evidently decreased by about 2.5 times through 100 years of exposure time, probably due to the weakened convection effects. Simultaneously, enhanced convection effects in terms of chloride build-up and permeation near the surface may impose exponentially increasing chloride diffusivity when humidity level decreases below 80%. This behavior is similar to experimental observations in [48], where the drop of external humidity levels from 75% to 50% leads to a notable increase in chloride diffusivity of concrete after wet/dry cycles.

According to Figure 6b, surface chloride concentration determined through extrapolating to the concrete surface increases with time and at the identical time generally increases with decreasing humidity level, except for the periods below 1 year, due to the negative value associated with the logarithm function. As the rate of moisture evaporation decreases with time, the growth of surface chloride concentration is initially promoted and later becomes gentle. This result is consistent with in situ investigations using the same logarithm function on the time-dependent surface chloride evolution [49]. As to the second point, surface chloride concentration at the identical exposure time increases with decreasing ambient humidity level. In particular, transient growth of surface chloride concentration was observed from 70 to 60% humidity level, indicating that the chloride contents at 100 years increase from 2.19 to 3.87% by weight of cement. This behavior may be due to the non-linearly increasing vapor permeability as non-saturated porosity increases [7]. Additionally, it can be related to the evolution of chloride concentration on the concrete surface being promoted as daily drying duration or tide height increases to certain levels [19,49].

As for the deviation of the numerical solutions from the diffusion model in Equation (24), the value increases with decreasing ambient humidity level from the saturation to 60%. This deviation would be due to a variation in the rate of moisture flow with penetration depth, making chloride profile steeply increased near the surface and sharply decreased through the deeper depth, which subsequently causes less accuracy in fitting results with the diffusion model. However, to date, any analytical models describing the chloride diffusion under cyclic exposure conditions have not been precisely determined to represent the sole diffusion behavior. In this respect, apparent diffusivity, depicted in Figure 6a, would represent the overall chloride diffusion and convection rate. Similarly, surface chloride concentration, depicted in Figure 6b, would represent the rate of chloride build-up near the drying surface due to the wick action.

## 4. Validation of Transport Model

### 4.1. In Situ Investigation

**Figure 7:**
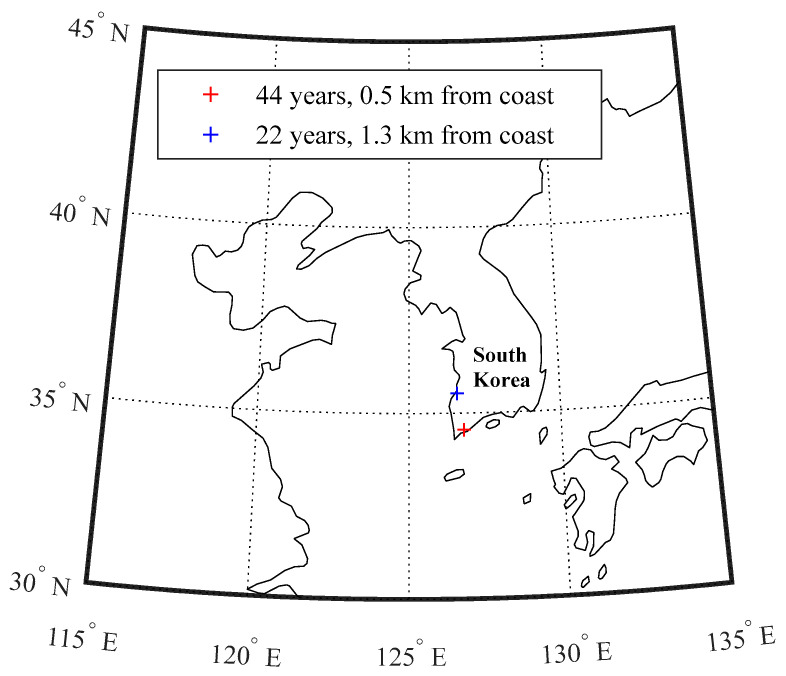
Geographical locations of two concrete bridges for in situ data acquisition.

The applicability of the coupled transport model proposed herein was verified using in situ data obtained from two concrete bridges built near the west and south coastlines in South Korea, as shown in Figure 7. The climatic conditions at those minimum and maximum values are summarized in Table 2. During low tide, core samples with 50 mm of length and 100 mm of diameter were obtained on two different aged structures, presumed to be 22 and 44 years at the measurement time. The sampling positions in both piers were determined at mean sea level with annual variation up to 0.13 m, presumably corresponding to 12 h of wet and dry per day. Those samples were then analyzed in the laboratory to determine total chloride profiles with 4–6 mm of increments near up to 50 mm of penetration depth using a conventional method (i.e., acid-soluble chlorides by 2.0 M HNO_3_). Due to the difficulty in considering a variation in surrounding environments from day to day, constant input values for specifying the boundary condition were used in the simulation, as given in Table 3. Both concrete piers are assumed to be made with 350–400 kg/m^3^ of cement content at 0.45 *w*/*c*, which equals the concrete properties with 10% of water-filled porosity and 2–8% of C_3_A content [47]. The corresponding input values for the pore structure and chloride binding capacity of the concrete are given in Table 4.

### 4.2. Simulation of Chloride Profile to In Situ Structure

**Figure 8:**
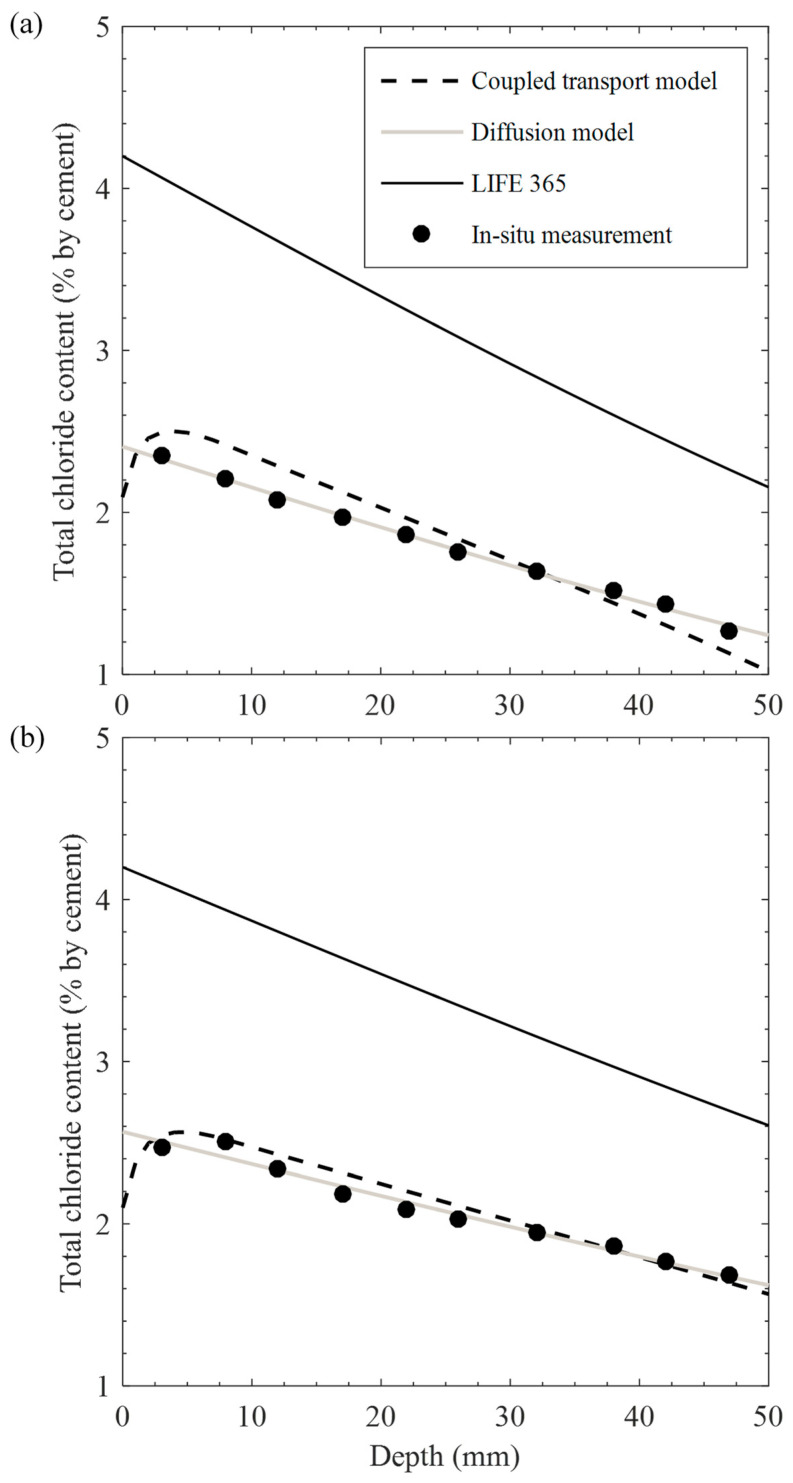
Calculated chloride profiles obtained from proposed numerical model and LIFE 365 together with diffusion model fitted to in situ data for (**a**) 22-year- and (**b**) 44-year-old concrete structures.

The chloride profile was simulated using the coupled transport model proposed in this study based on the above considerations. Simultaneously, it was compared with the result from LIFE 365 as a widely used software for service life prediction of steel-reinforced structures [50], considering that a single driving force of chloride diffusion is obtained at the identical exposure condition. Both chloride profiles are depicted in Figure 8 with the in situ data. The diffusion model in Equation (24) was used to determine the diffusion parameters (i.e., *C_s_* and *D_a_*) through fitting the in situ data. For the simulation of LIFE 365, the maximum chloride content accumulated at the concrete surface was assumed to be 0.8% by weight of concrete (4.2% by weight of cement). As for the time-dependent chloride diffusivity in LIFE 365 with a similar form in Equation (24), empirical correlations for OPC concrete with 0.45 *w*/*c* were used to determine the reference diffusivity at 28 days and its decay constant, accounting for 1.05 × 10^−11^ m^2^/s and 0.2, respectively. As seen in Figure 8, the chloride profile calculated from the coupled transport model shows a reasonable correlation to the in-situ data, revealing 0.83 and 0.93 of determinant coefficients for 22 and 44 years, respectively. This numerical analysis can also cover the low concentration at the surface (i.e., 2.1% by weight of cement), resulting from the intermittent exposure of chloride source with concentration build-up near the surface. However, despite the time dependency on the chloride diffusion rate, the results from LIFE 365 are far from the in situ data due to the higher surface chloride content, in which the condition was set up in the case of the tidal zone in New York achieved after 1 year of exposure. Furthermore, the diffusion coefficients in LIFE 365 decrease from 3.37 × 10^−12^ m^2^/s for 22 years to 2.94 × 10^−12^ m^2^/s for 44 years, indicating a 12.7% decrement, which is about two times more than that for the diffusion model from 22 to 44 years. In turn, this considerable reduction in chloride diffusivity with time in the software may also lead to low consistencies with the in situ data even if the constant surface concentrations in both exposure periods are properly set within those from the diffusion model, accounting for 2.40% and 2.57% by weight of cement for 22 and 44 years, respectively. 

The differences between the in situ observation and those for LIFE 365 with a single diffusion analysis would mainly be attributable to the absence of moisture transport in the prediction tool. However, the concentration distribution through the inner concrete depth tends to follow diffusion rather than convection, as described in Section 3.3. For example, in Figure 8, the chloride profile obtained by fitting the in situ data with the diffusion model provides determinant coefficients over 0.98, except 3–5 mm of the distance from the surface. Hence, for better prediction with a single diffusion analysis, surface chloride concentration and chloride diffusivity should reflect the time dependency on the material and environmental condition. Provided that surface chloride concentration with time dependency is properly determined based on the numbers of in situ investigations [49], chloride diffusivity to track the chloride profile over the diffusion-controlled zone will be an essential parameter in the prediction. For determining the chloride diffusivity at saturated [20,32] and non-saturated [21,51] conditions, pore structure in terms of porosity and pore connectivity should be considered. This study determined pore size distribution to obtain the tortuosity at interconnected capillary pores and thus effective chloride diffusivity with the ink-bottle effect. Though time dependency on the pore size distribution was not considered in this study, cement hydration continuously densifies the microstructure for chloride transport with time. When the degree of hydration reaches its threshold value, depercolation of the pore system [32,36] considerably limits the ionic mobility even in a non-saturated medium [51]. Nevertheless, the simulation result from the coupled transport model with a chloride diffusivity invariant with time (Equation (20)) shows satisfactory predictions to the in situ data. However, in this case, time-dependent convection effect varying with moisture distribution to influence the ionic penetration with time was reflected in the simulation. As seen in Figure 6a, chloride diffusivity depends largely on the convection effects that change with exposure time and external humidity conditions. In this respect, the chloride diffusivity should also include the convection effects associated with both moisture and chloride transport if one uses a simple diffusion model for the service life prediction under the cyclic environments. Otherwise, it is better to use a numerical solution for the analysis.

## 5. Conclusions

In this study, a coupled transport model under cyclic exposure to chloride was developed, taking into account pore structure effects on both the moisture and chloride transport rates in concrete. Based on the simulation results, the following conclusions can be drawn:

(1) Pore size distribution was determined to provide pore structure parameters for mass transport coefficients. By reconstructing pore size distribution with hysteric behavior between vapor adsorption and desorption, it was found that pore size with 4.0 nm in radius shows maximum connection probability to large pores. By applying the fraction of entrapped water content to the composite medium model, the effective chloride diffusivity covering gel to capillary pores can be derived.
(2) Moisture distributions under constant and cyclic exposure conditions were calculated based on the proposed numerical solution. As a result, moisture tends to evaporate toward the drying surface to reach equilibrium through inner material depth, which is more quickly achieved by cyclic exposure conditions, and the inner moisture level at equilibrium increases with the ambient humidity level.
(3) Under the intermittent exposure of salt solution, the influence of moisture level on the behavior of chloride transport with concentration profiles varying with ambient humidity conditions was assessed. The chloride transport is particularly dominant at a 60% humidity level, showing a higher rate of chloride ingress with time, which also entails a higher risk of corrosion as critical concentration reaches the assumed steel depth. However, the rates of chloride transport at medium humidity levels (e.g., 70 and 80%) are relatively low compared to the saturation one due to the less-significant convection effects and reduced chloride diffusivity.
(4) Analytical solutions of chloride diffusion parameters as functions of time and ambient humidity level were determined. As a result, both chloride diffusivity and surface chloride concentration are significantly dependent on the moisture level, implying that convection is a decisive factor in analyzing chloride diffusion under cyclic environments.
(5) Chloride profiles obtained from the proposed transport model, analytical solution, and LIFE 365 were compared, indicating that correlations with the in situ data were satisfactory in using the proposed model and highest for the analytical solution with optimized diffusion parameters. The results are far from those for LIFE 365, giving poor correlations. This implies that convection effects on ambient conditions and diffusion properties on concrete pore structure are essential in predicting the service life under cyclic environments.

## Figures and Tables

**Table 1:** Material parameters derived from pore structure model.

Property	Notation	Gel Pores	Capillary Pores	
		*i* = 1	*i* = 2 (Small)	*i* = 3 (Large)
Pore distribution	$\emptyset_{i}$	0.03	0.05	0.12
	$B_{i}^{-1}$ (nm)	0.75	3.99	50.47
Tortuosity	$F\left(r_{con}\right)$	-	0.26	
	τ	8.94		
Effective pores	y	0.47		
	$D_{e}$ (m^2^/s)	2.09 × 10^−12^		

**Table 2:** Regression analysis on numerical solution varying with relative humidity using analytical solutions.

Parameter	Relative Humidity at External Condition (%)								
	60	65	70	75	80	85	90	95	100
MSE (%^2^)	0.0759	0.0274	0.0180	0.0143	0.0123	0.0110	0.0101	0.0092	0.0083
m	0.4780	0.2032	0.1209	0.0840	0.0646	0.0538	0.0479	0.0442	0.0427
k (%/ln(yr))	0.0055	0.0883	0.1239	0.1407	0.1483	0.1508	0.1493	0.1445	0.1331

**Table 3:** Environment conditions for in situ data and corresponding model input values.

	Temperature (°C)	Humidity (%)	Tide Level (m)
Minimum	5.5	24	3.5
Maximum	28.9	97	12.0
Input	20.0	70	7.7

**Table 4:** Assumed properties for numerical solution of chloride penetration in concrete bridges.

Property	Notation	Gel Pores	Capillary Pores	
		*i* = 1	*i* = 2 (Small)	*i* = 3 (Large)
Pore structure	$\emptyset_{i}$	0.09	0.01	0.09
	$B_{i}^{-1}$ (nm)	0.75	3.91	51.23
	$F\left(r_{con}\right)$	-	0.37	
	τ	6.22		
	$D_{e}$ (m^2^/s)	3.28 × 10^−12^		
Chloride binding	α	1.15		
	β	0.28		

## Data Availability

Data is contained in this article.
